# Early Outcomes and Complications of Skin Graft Reconstruction for Complex Wounds at a Tertiary Hospital: A Single-Centre Study in Oman

**DOI:** 10.3390/diseases14080286

**Published:** 2026-08-10

**Authors:** Ali Abduwani, Abdullah Al Lawati, Ruai Al Abri, Reem Al Mayyahi, Hoor Al Barhi, Shatha Al Hussaini, Moath Shummo, Hanan Al Lawati, Nawaf Al-Muqaimi, Srijit Das

**Affiliations:** 1 Department of Human and Clinical Anatomy, College of Medicine and Health Sciences, Sultan Qaboos University, Al Khoudh, Muscat 123, Oman; s136668@student.squ.edu.om (A.A.); s136510@student.squ.edu.om (R.A.A.); s137566@student.squ.edu.om (R.A.M.); 2 Plastic Surgery Unit, Department of Surgery, Sultan Qaboos University Hospital, Al Khoudh, Muscat 123, Oman; s131482@student.squ.edu.om (A.A.L.); m.shummo@squ.edu.om (M.S.); nawaf.m@squ.edu.om (N.A.-M.); 3 Department of Medicine, Royal College of Surgeons in Ireland, D02 YN77 Dublin, Ireland; hoorbadarsaifa21@rcsi.com (H.A.B.); shathaalhussaini21@rcsi.com (S.A.H.); 4 Pharmacy Program, Oman College of Health Sciences, Muscat 113, Oman; allawati@ualberta.ca

**Keywords:** skin graft, complex wounds, graft take, wound healing, postoperative complications

## Abstract

**Background:** Complex wounds present significant reconstructive challenges owing to impaired healing, infections, and tissue loss. Skin grafting remains a widely used reconstructive technique; however, outcome data from the Gulf region are limited to a few studies. This study aimed to evaluate early graft outcomes and complications following skin graft reconstruction for complex wounds at a tertiary hospital. **Methods:** A retrospective, hospital-based study was conducted at Sultan Qaboos University Hospital (SQUH), including all patients who underwent split-thickness or full-thickness skin grafting between 2023 and 2025. Demographic, clinical, surgical, and wound-related data were collected. The main outcomes were graft take on postoperative days 5–7, time to complete re-epithelialization, and the occurrence of postoperative complications. **Results:** A total of 50 patients were included; 72% were males, with a median age of 45.5 years. Diabetes mellitus was present in 34% of patients. Trauma (28%) and infection/necrotizing causes (22%) were the most common etiologies of wounds. Complete graft take (>90%) was achieved in 90% of the patients, whereas 10% had partial graft take. The median time to complete re-epithelialization was 13 days. Complications were infrequent and minor, including postoperative infection (8%), hypertrophic scar formation (6%), and wound dehiscence (2%). No significant differences in healing time were observed between the diabetic and non-diabetic patients. **Conclusions:** In this descriptive institutional cohort, a combined protocol of wound-bed preparation, skin graft reconstruction, and postoperative NPWT-assisted stabilization was associated with favorable early graft take and fewer complications. These findings should be interpreted cautiously because of wound heterogeneity, unavailable wound size measurements, and limited subgroup sizes. Larger prospective studies are needed to evaluate the predictors of graft success.

## 1. Introduction

Complex wounds are challenging soft-tissue defects that frequently require advanced reconstructive strategies. Although no universal definition exists, complex wounds are generally defined as those that fail to progress through the normal healing process and/or exhibit features that impair recovery, such as infection, necrosis, compromised tissue perfusion, or extensive tissue loss [1,2]. These wounds may arise from trauma, postoperative complications, chronic ulcerations, or severe infections and may expose vital structures, such as tendons or bones, significantly complicating reconstruction and delaying wound healing [1,3]. Complex and chronic wounds pose a significant healthcare burden. In the United States, they affect approximately 10.5 million Medicare beneficiaries and generate an annual healthcare cost of approximately $22.5 billion [4]. Stable wound coverage is essential for preventing infection, restoring function, and promoting timely wound healing.

Skin grafting remains one of the most commonly used reconstructive techniques for managing soft-tissue defects when primary closure is impractical and local or free flap reconstruction is unnecessary or unstable [5]. The two main types of skin grafts used in reconstructive surgery are split-thickness skin grafts (STSGs) and full-thickness skin grafts (FTSGs). STSGs include the epidermis and a part of the dermis and are widely used because they can cover larger surface areas, as surgeons can mechanically expand the harvested graft, most commonly by meshing or Meek micrografting, which increases the recipient site coverage while reducing donor-site skin requirement and has higher graft survival rates [6,7,8]. However, these grafts are associated with increased secondary contraction and less favorable cosmetic outcomes [9]. In contrast, full-thickness skin grafts include the entire dermal layer, resulting in better cosmetic outcomes and less contraction. However, FTSGs have a greater risk of graft failure, are more limited in the size of the defect and choice of donor site, and require a well-vascularized recipient bed [5,6,9,10]. Regardless of graft type, successful grafting depends largely on the vascular quality of the recipient bed, as graft survival relies on revascularization, commonly referred to as graft take [9].

A combination of patient-, wound-, and treatment-related factors influences skin graft outcomes. Patient characteristics, such as age, body mass index (BMI), diabetes mellitus, glycemic control, and smoking status, have been associated with impaired wound healing and graft survival [11,12]. The condition of the wound bed is a critical determinant of graft success. Effective grafting requires a clean, uncontaminated, and well-vascularized recipient site with adequate granulation tissue. The survival of a skin graft depends on revascularization, beginning with plasmatic imbibition, followed by capillary ingrowth and vascular anastomosis in the recipient bed. Consequently, inadequate vascularity, necrotic tissue, or poor granulation can compromise graft adherence and reduce graft uptake [13]. Treatment-related factors may also affect outcomes. Adjunctive therapies, such as negative-pressure wound therapy (NPWT/VAC), have been shown to improve graft stability and enhance graft take by increasing local perfusion, reducing edema, and promoting wound healing [14,15]. Additional strategies, including dermal substitutes and appropriate graft fixation techniques, may further optimize the wound environment and improve graft integration [14,15].

Although several studies have examined the predictors of graft success, the reported outcomes remain different due to the multifactorial nature of graft uptake. Much of the available evidence originates from Western institutions and high-volume tertiary centers in the West. Data on surgical outcomes in the Gulf region are limited, and there is a notable lack of published institutional data on skin graft outcomes for complex wounds in Oman. Regional differences in patient demographics, high prevalence of metabolic comorbidities, trauma patterns, and variations in surgical practice may influence graft outcomes. In the Gulf region, distinct demographic and clinical characteristics may further influence wound healing and reconstructive outcomes [16,17]. Therefore, generating local evidence is essential for better understanding outcome patterns, guiding clinical decision-making, and optimizing reconstructive strategies in the regional healthcare context of the study.

This study aimed to descriptively evaluate patient characteristics, wound features, reconstructive practices, early graft outcomes, and postoperative complications following skin graft reconstruction at the Sultan Qaboos University Hospital. Exploratory comparisons were performed to identify potential outcome patterns rather than to establish independent predictors of graft success. This study provides institution-specific baseline data on local reconstructive practices.

## 2. Materials and Methods

### 2.1. Study Design and Setting

The retrospective, hospital-based study was conducted at Sultan Qaboos University Hospital, Muscat, Oman, including patients who underwent skin graft reconstruction between 2023 and 2025.

### 2.2. Patient Selection

All patients who underwent skin graft reconstruction for complex wounds at SQUH during the study period were included in the study. For this study, “complex wounds” were operationally defined as heterogeneous soft-tissue defects that were not amenable to primary closure and required skin graft reconstruction after appropriate wound-bed preparation. This reconstructive category included traumatic, surgical/procedure-related, burn-related, infective/necrotizing, diabetic/metabolic, and oncological wounds. These etiological groups have been reported separately and are not considered biologically equivalent. However, exposed tendons or bones were not considered as isolated indications for direct skin grafting. In these cases, grafting was performed only after appropriate wound-bed optimization, including debridement, NPWT, and, when required, MatriDerm^®^ application until the exposed structures were covered by healthy granulation tissue or a suitable vascularized wound bed. Both split-thickness skin grafts (STSGs) and full-thickness skin grafts (FTSGs) were considered in this study. Patients were included if their complete medical records and postoperative follow-up data were available for review. Patients were excluded if their medical records were incomplete, if they underwent surgery in departments other than Plastic Surgery, or if they were lost to postoperative follow-up.

### 2.3. Wound Preparation and Postoperative Care

Before definitive reconstruction, the wounds underwent serial debridement as required and were managed with negative-pressure wound therapy (NPWT) at 120 mmHg. Dressings were routinely changed every 72 h until the wound bed was deemed suitable for grafting purposes. In wounds with exposed tendons or bones, skin grafting was not directly applied to the bare avascular structures. These wounds underwent further wound-bed preparation using NPWT and, in selected cases, MatriDerm^®^, until adequate granulation tissue or a suitable vascularized surface was achieved. In selected patients with exposed tendons or bones, a dermal substitute (MatriDerm^®^; MedSkin Solutions Dr. Suwelack AG, Billerbeck, Germany) was used as part of the reconstructive strategy. In these cases, a 1 mm MatriDerm^®^ was applied as a bilayer dermal substitute to optimize the wound bed and facilitate subsequent grafting. Split-thickness skin grafts (STSGs) were applied as meshed grafts with a 1:1.5 expansion ratio. Following skin graft application, NPWT was applied over the graft at 100 mmHg to promote graft adherence and minimize shear forces. The dressing was maintained for seven days postoperatively, after which graft uptake was assessed. Accordingly, the reported outcomes reflect a combined reconstructive pathway involving debridement, wound-bed optimization, skin grafting, and postoperative NPWT-assisted graft stabilization rather than the effect of skin grafting in isolation.

### 2.4. Data Collection

Data were extracted retrospectively from the hospital information system (TrakCare) at SQUH using a standardized data collection sheet reviewed and approved by the supervisor (a plastic surgery consultant). Demographic data included age, sex, and body mass index (BMI). The clinical characteristics included the presence of comorbidities such as diabetes mellitus, peripheral arterial disease, smoking status, hypertension, and other chronic conditions. Diabetes status was recorded as a binary variable (no = 0, and yes = 1).

Wound-related characteristics included etiology, wound duration before grafting, microbiological findings from wound cultures, wound depth, presence of exposed critical structures, and condition of the wound bed, including infection, necrosis, and granulation. Wound-bed characteristics were not mutually exclusive, and more than one feature could be present in a single wound bed. Preoperative wound management variables included the use of negative-pressure wound therapy (NPWT), also known as vacuum-assisted closure (VAC) therapy, with the number of VAC dressing changes recorded, where applicable.

Operative variables included graft type, anatomical site of graft placement, donor site, use of dermal substitutes such as MatriDerm^®^, and graft fixation methods. The choice between split-thickness and full-thickness skin grafting was based on anatomical location, wound characteristics, and consultant surgeon assessment rather than the random allocation of patients. In our cohort, FTSG was reserved for facial wounds, where improved color and texture match and reduced secondary contraction were considered important factors. STSG was used for most non-facial wounds, particularly larger wounds requiring broader surface coverage or meshing techniques. Postoperative outcomes were assessed in the early postoperative period. Graft take was evaluated between postoperative days 5 and 7 and recorded as the percentage of graft survival. Graft take was categorized as complete (>90%) or partial (<90%) for descriptive analysis. The time required for complete epithelialization was recorded in days. Additional postoperative outcomes included hypertrophic scar formation and postoperative complications, such as graft necrosis, hematoma formation, surgical site infection, and wound dehiscence. Complications were classified as minor or major. Minor complications required conservative or non-surgical management, whereas major complications required surgical intervention, intensive medical management, or hospital readmission. When patients experienced multiple complications, the classification was based on the most severe complication. The follow-up duration was calculated from the date of skin graft reconstruction to the last documented plastic surgery clinic review date.

### 2.5. Outcomes

The primary outcome was graft take, defined as the percentage of viable grafts observed between postoperative days 5 and 7 [12]. The secondary outcomes included postoperative complications, time to complete epithelialization, and hypertrophic scar formation.

### 2.6. Statistical Analysis

Data were entered and analyzed using the IBM Statistical Package for the Social Sciences (SPSS) software (version 26; IBM Corp., Armonk, NY, USA). Continuous variables, including age, number of VAC dressing changes, and time to complete re-epithelialization, are presented as medians with interquartile ranges (IQR). Categorical variables were expressed as frequencies and percentages.

The associations between the categorical variables were assessed using Fisher’s exact test. The time to complete re-epithelialization was analyzed using Kaplan–Meier survival analysis, with comparisons made using the log-rank test. The median healing times between groups were compared using the Mann–Whitney U test. A two-sided *p*-value of <0.05 was considered statistically significant. Given the limited cohort size and only five partial graft-take events, all association analyses were considered to be exploratory. No multivariable predictor modelling was performed, and the study was interpreted primarily as a descriptive institutional cohort.

### 2.7. Ethical Approval

Ethical approval was obtained from the Medical Research Ethics Committee (MREC) at the College of Medicine and Health Science, Sultan Qaboos University, Muscat, Oman. Due to the retrospective nature of the study, informed consent was not required. Patient confidentiality was maintained throughout the study, and all extracted data were anonymized before statistical analysis.

## 3. Results

**Figure 1:**
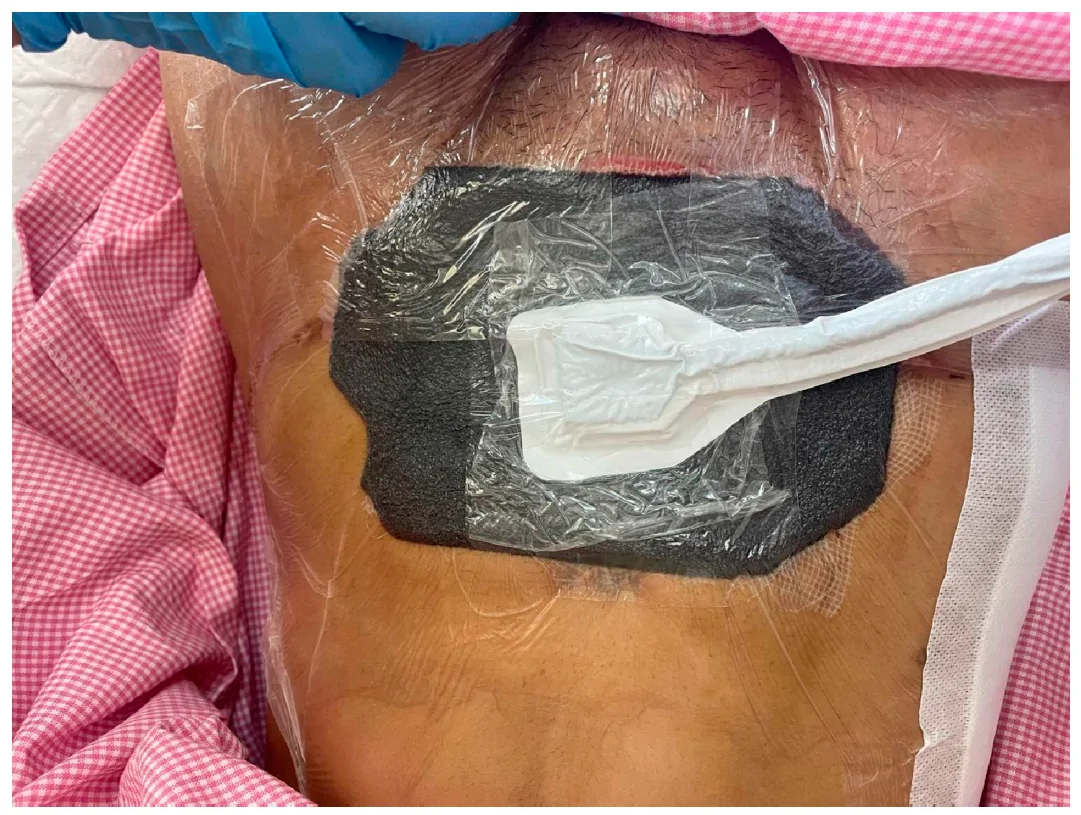
A vacuum-assisted closure dressing was applied to promote wound healing.

**Figure 2:**
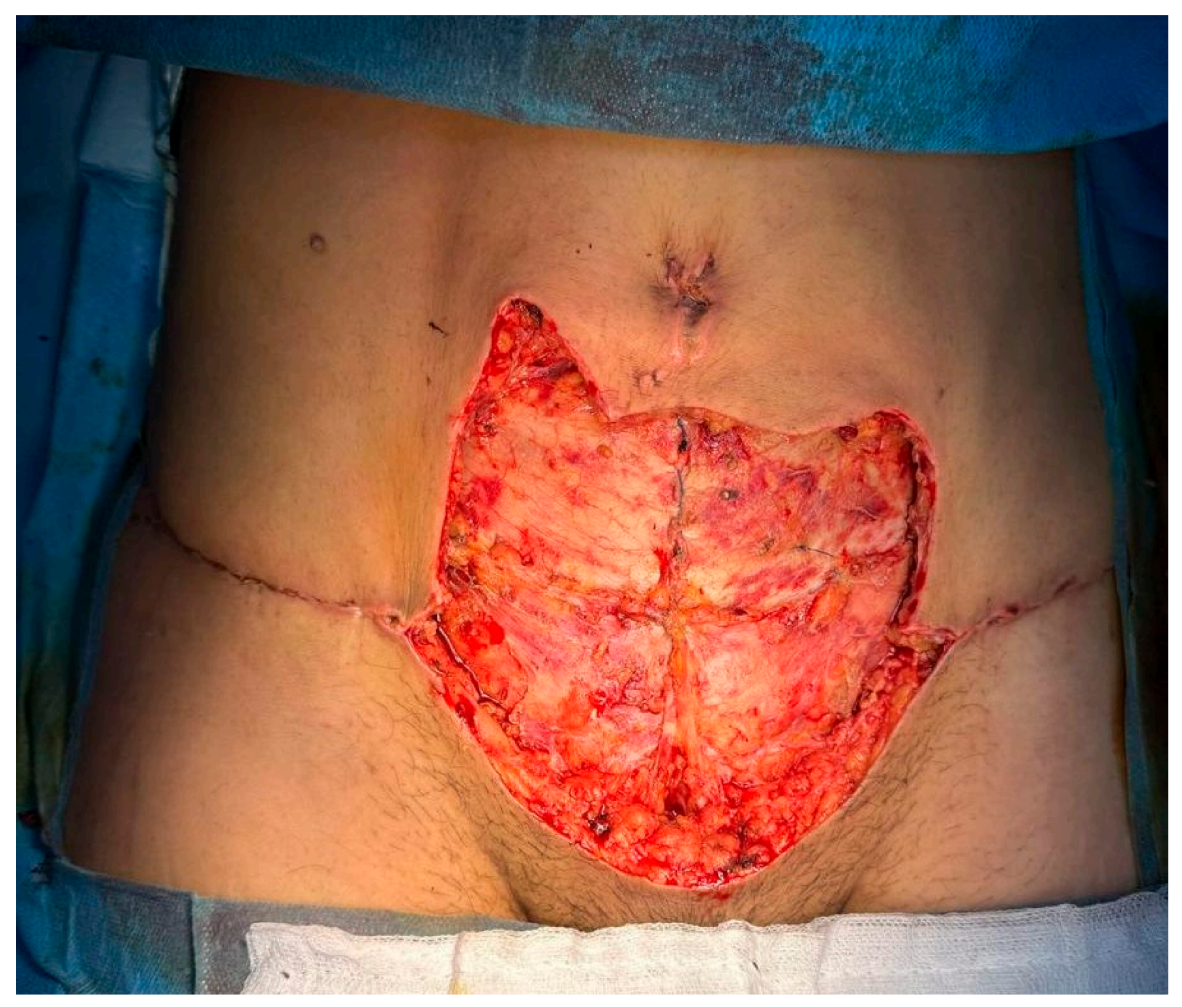
Intraoperative appearance of the lower abdominal wound, demonstrating a healthy wound bed prepared for definitive reconstruction.

A total of 50 patients were included in the study, comprising 36 males and 14 females. The median (interquartile range [IQR]) age was 45.5 (34.3) years. Comorbidities included diabetes mellitus in 34%, hypertension in 26%, and dyslipidemia in 14% of patients. Hematological malignancies were present in 10% of patients, and 4% had sickle cell disease. The demographic characteristics of the study population are shown in Table 1. The median (IQR) wound duration before reconstruction was 28.50 (39) days. The wound etiology is detailed in Table 2, with trauma (28%) and infective or necrotizing causes (22%) being the most common causes. Most patients (68%) had partial thickness wounds, whereas 32% had full thickness wounds. In 4% of patients, the tendon was exposed, whereas in 16%, the bone was exposed. The median (interquartile range [IQR]) number of VAC dressing changes before reconstruction was 1 (0–8). Figure 1 shows the application of the vacuum-assisted closure dressing. Preoperatively, 56% of wounds were infected, 46% showed evidence of necrosis, and granulation was observed in 24% of cases. Among the 28 infected wounds, the most frequently isolated pathogens were *Pseudomonas* species (including *P. aeruginosa* and *P. stutzeri*) (n = 6, 21.4%), followed by *Klebsiella pneumoniae* (including MDR strains) and *Escherichia coli* (including ESBL-producing strains), each isolated in four cases (14.3%), MRSA in three cases (10.7%), and *Streptococcus pyogenes* in three cases (10.7%). Fifteen patients (53.6%) were treated with systemic antibiotic therapy. Antibiotic therapy was guided by culture and sensitivity results when available and clinically indicated. Figure 2 shows the intraoperative lower abdominal wound.

**Figure 3:**
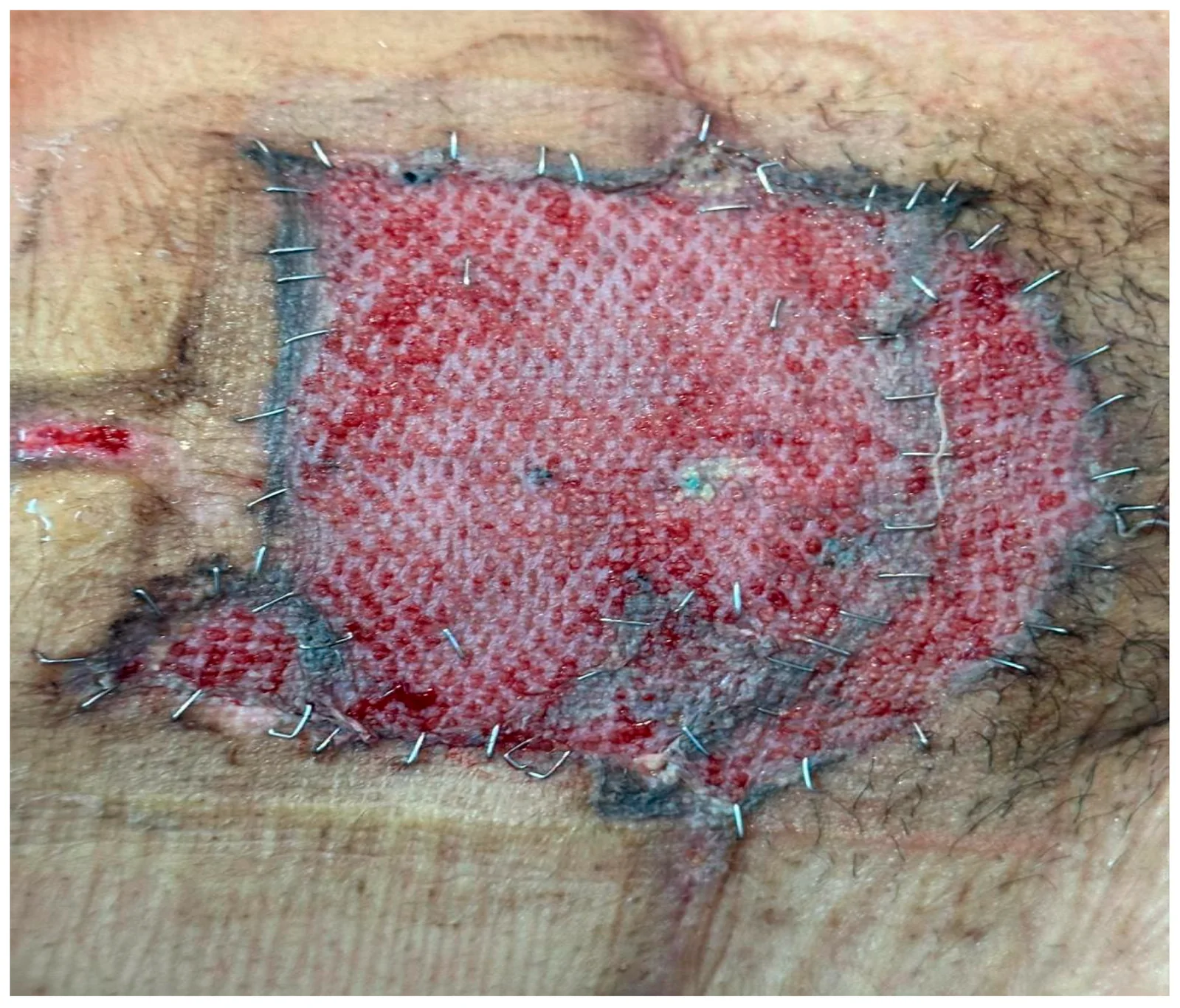
One-week postoperative appearance following application of a meshed skin graft.

**Figure 4:**
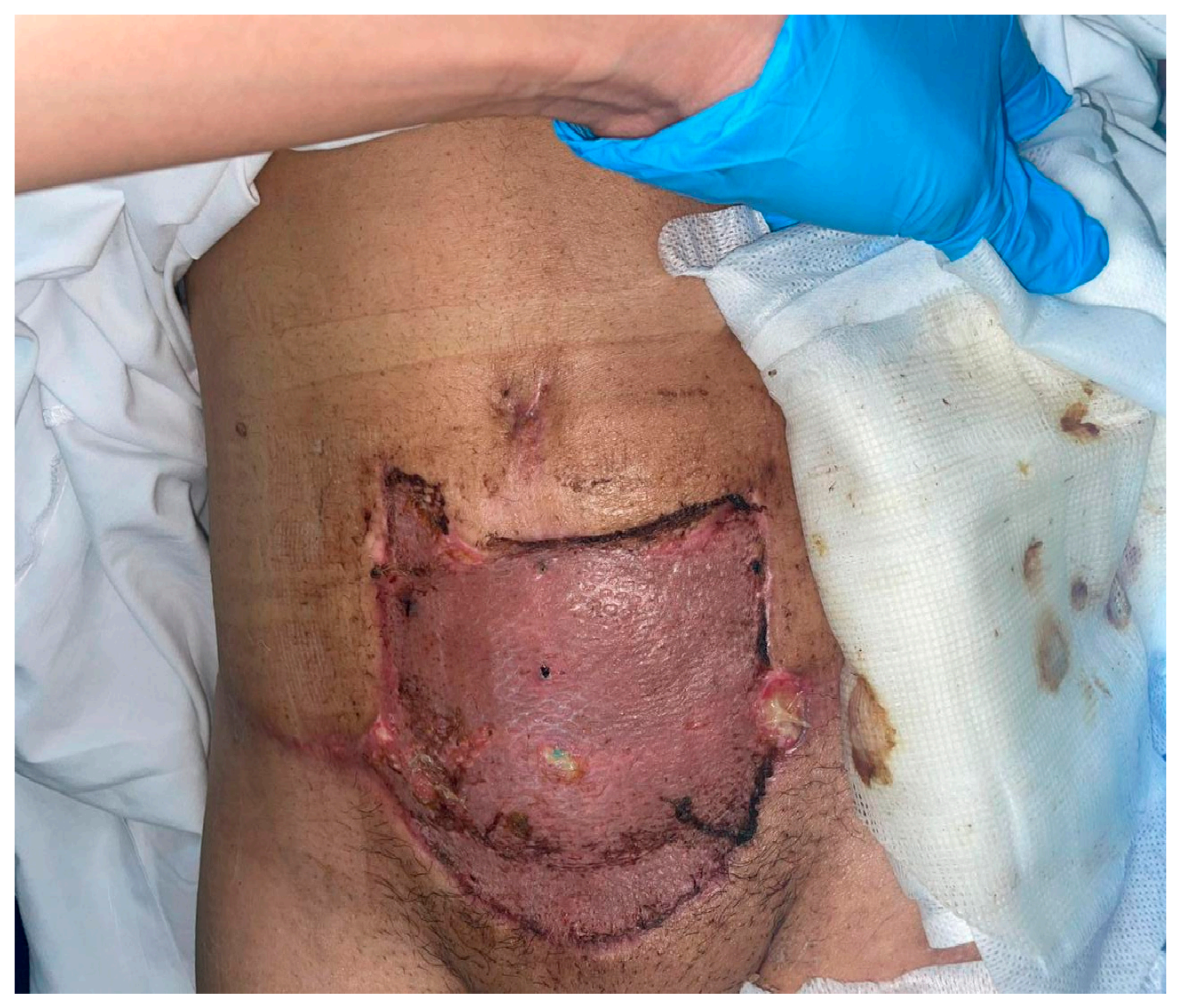
One-month postoperative appearance following application of a meshed skin graft.

**Figure 5:**
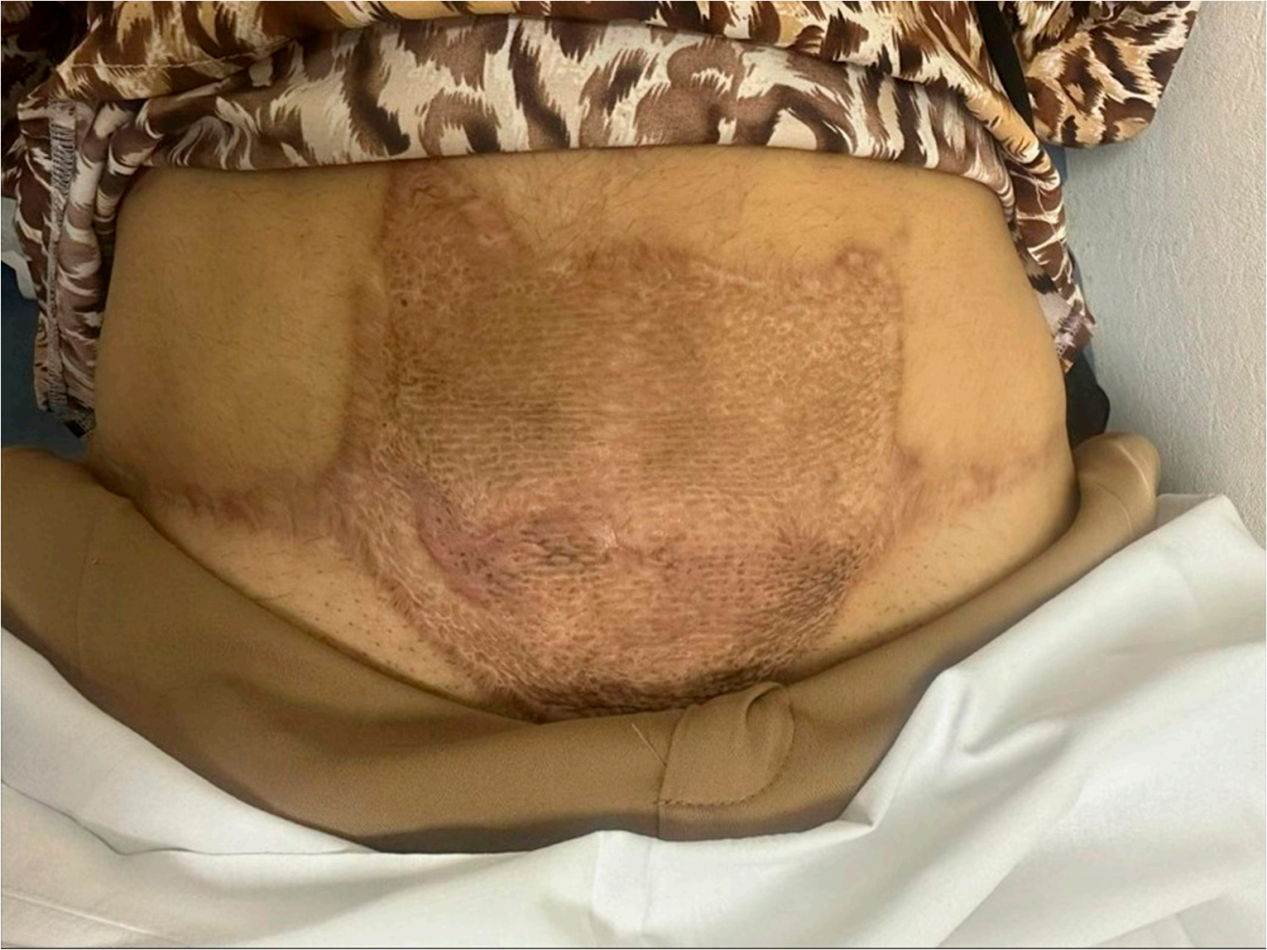
One-year postoperative appearance following application of a meshed skin graft.

Split-thickness skin grafts were used in 76% of the cases, whereas 24% underwent full-thickness skin grafting. The thigh was the most common donor site (86%), followed by the arm (8%), groin (2%), lower abdomen (2%), and supraclavicular region (2%). MatriDerm^®^ was used in five patients (10%) as part of the wound-bed preparation in selected cases. Given the small and selectively treated subgroup, no comparative conclusions regarding its effectiveness were made. At 5–7 days postoperatively, complete graft take (>90%) was observed in 90% of cases, whereas partial graft take was noted in 10%. The association between graft uptake on days 5–7 postoperatively and the different factors is shown in Table 3. The median (interquartile range [IQR]) time to complete re-epithelialization was 13 (18) days. Hypertrophic scar formation occurred in 6% of patients, postoperative infection in 8%, and wound dehiscence in 2% of patients. Figure 3, Figure 4 and Figure 5 show the postoperative appearance at one week, one month, and one year, respectively.

**Figure 6:**
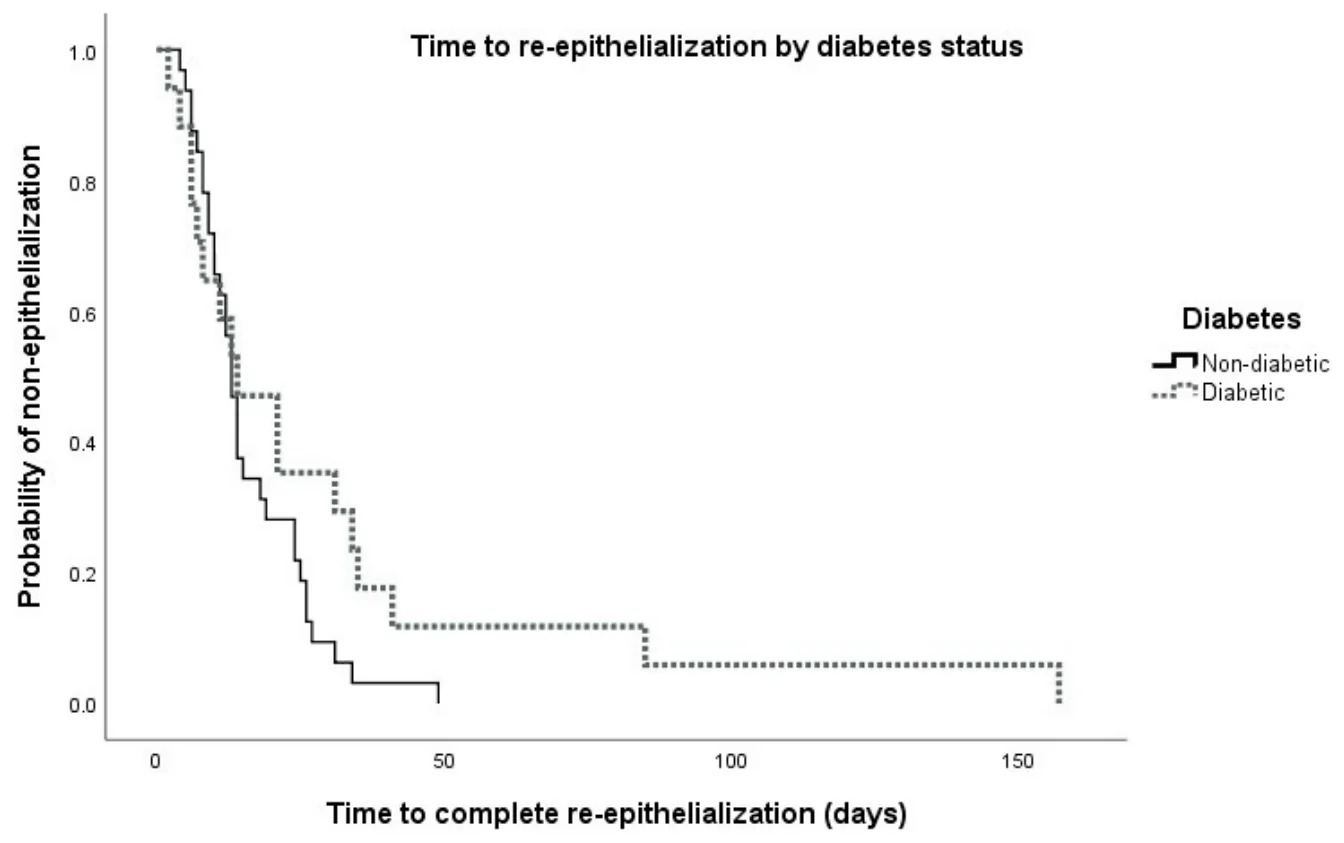
Kaplan–Meier curve showing time to complete re-epithelialization by diabetes status, demonstrating similar healing trajectories between groups, with a small subset of diabetic patients exhibiting prolonged healing (log-rank *p* = 0.127).

The median (interquartile range [IQR]) time to complete re-epithelialization was 12 (28) days in pediatric patients and 13.50 (18) days in adults, with no significant difference between the groups (*p* = 0.761). Similarly, no significant difference was observed between the diabetic and non-diabetic patients, with median (IQR) times of 14 (28) and 13 (15) days, respectively (log-rank *p* = 0.127). Figure 6 shows similar healing trajectories, with a small subset of patients with diabetes exhibiting prolonged healing. The median follow-up duration was 124 days (IQR: 169 days), with a maximum duration of 512 days.

Patients with preoperative wound infection had a longer time to complete re-epithelialization (14 [19] days) than those without infection (13 [15] days), although this difference was not statistically significant (*p* = 0.511). In contrast, wounds with granulation tissue required a longer time to achieve complete re-epithelialization (16.5 [27] days) compared to non-granulating wounds (12 [18] days). This finding should be interpreted cautiously, as granulating wounds may represent larger, deeper, or more complex wounds that require a longer period to achieve full epithelial coverage.

Necrotic wounds demonstrated a shorter time to complete re-epithelialization (10.5 [12] days) compared to non-necrotic wounds (15 [20] days). However, this result should not be interpreted as a beneficial effect of necrosis. This may instead reflect effective pre-grafting debridement, wound selection, differences in wound size or depth, or other unmeasured confounding factors.

## 4. Discussion

This study descriptively evaluated the early outcomes following skin graft reconstruction in a heterogeneous institutional cohort. Complete graft take was observed in 90% of cases, with a median time to complete re-epithelialization of 13 days and few complications reported. These outcomes should not be attributed to skin grafting alone, because NPWT was used before reconstruction to optimize the selected wound beds and again after graft placement as a postoperative bolster. Therefore, the findings represent an integrated protocol involving debridement, wound-bed preparation, skin grafting, and NPWT-assisted graft stabilization. Although the wounds shared a common reconstructive endpoint, their different etiologies carried distinct biological and surgical considerations.

The graft success rate in this study was comparable to that reported in the literature. Previous reviews and clinical studies have shown that graft take rates are commonly around 85–90% in well-prepared wounds [14,16,17,18,19,20,21]. For instance, a study by Kemp Bohan et al. reported a mean graft take of 86.9% ± 19.5% [22]. Consequently, other prospective studies have considered a successful graft take as ≥ 95% viability [12]. In more complex cases, such as burns, a successful graft take is considered one with viability > 70% [23]. Conversely, another systematic review reported that graft success is multifactorial, noting that there is no significant relationship between graft outcomes and factors such as burn severity, antibiotic use, wound etiology, or comorbidities [24]. This similarity likely reflects adherence to the fundamental determinants of graft success, including adequate vascularization, wound-bed preparation, and infection control [21]. In addition, split-thickness skin grafting was used in most patients in this cohort, consistent with routine reconstructive practice and likely contributing to the favorable early outcomes observed [19]. This aligns with previous studies, which describe split-thickness skin grafting as the most preferred and gold standard technique for large skin defects [21,25]. Several studies have demonstrated that split-thickness skin grafts are associated with skin take rates that exceed 90% [26]. Additionally, a retrospective cohort study demonstrated that split-thickness skin grafts achieved significantly higher graft take rates (90%) compared to full-thickness grafts (72%) [20]. Moreover, split-thickness skin grafts are versatile, widely applicable, and effective in routine clinical practice, leading to better and faster recovery [27].

The present study did not demonstrate statistically significant associations between graft uptake and several traditionally recognized risk factors, including diabetes, preoperative wound infection, age, granulation tissue, and necrotic wounds. Although diabetes is widely reported to be associated with impaired wound healing and reduced graft survival [22,23], no significant association was observed in the present study. This finding should be interpreted with caution, as the relatively small sample size likely rendered the study underpowered to detect clinically meaningful associations between graft uptake and the mentioned variables. Consequently, the absence of statistically significant results may reflect insufficient statistical power, rather than a true lack of association.

Ramanujam et al. (2013) [28] reported that the time to healing of STSG was prolonged by 1.99 weeks in diabetic patients compared to non-diabetic patients. However, following stratification, no significant difference in healing time was observed between non-diabetic patients and diabetic patients without associated comorbidities, suggesting that the effect of diabetes may be influenced by the presence of additional systemic factors [28].

This may reflect effective perioperative optimization, including infection control, debridement, and wound-bed preparation, thereby reducing the early effects of systemic comorbidities on graft uptake. This may also reflect the limited statistical power of a relatively small single-center cohort. Similar findings in smaller studies suggest that when local wound conditions are well managed, they may play a more important role than systemic comorbidities in early graft success [19].

No significant differences in the time to complete re-epithelialization were observed between pediatric and adult patients or between diabetic and non-diabetic patients. However, these findings should be interpreted with caution, as the relatively small sample size may have limited the statistical power to detect potentially meaningful differences between groups. Patients with preoperative wound infection demonstrated a longer time to complete re-epithelialization, although this was not statistically significant. In contrast, wounds with granulation tissue required a longer time to achieve complete re-epithelialization. This finding should be interpreted as an association, rather than a causal relationship. Granulating wounds may have been larger, more complex, or more chronic at baseline, potentially contributing to the prolonged healing times.

Interestingly, necrotic wounds demonstrated a shorter time to complete re-epithelialization. This counterintuitive finding should be interpreted cautiously. Necrotic tissue is generally considered unfavorable for graft survival because it may impair graft adherence, vascular ingrowth, and wound-bed integration [13,22]. Therefore, the observed association should not be interpreted as evidence that necrosis improves healing. A more likely explanation is that wounds documented as necrotic underwent effective debridement and wound-bed optimization before being grafted. This finding may also reflect case selection, differences in wound size or depth, timing of reconstruction, or residual confounding that could not be fully assessed in this retrospective study design. Similarly, the longer healing time observed in granulating wounds may not indicate that granulation tissue itself delays healing but may instead reflect that these wounds were larger, deeper, or more complex at baseline.

Although several studies have examined the predictors of graft success, the reported outcomes remain different due to the multifactorial nature of graft uptake. The literature reports that patient-related factors, such as diabetes, obesity, bedridden state, nutritional status, stress, uremia, alcoholism, and smoking, influence outcomes [29]. However, the extent of grafting, timing of grafting, dressing type, topical gentamicin use, and debridement requirement had no significant effect on graft outcomes [30]. Furthermore, maintaining adequate hemoglobin (≥10 g/dL) is important for graft uptake, as oxygen delivery in the early phase of healing directly influences graft success [31]. Moreover, graft outcomes depend not only on patient and wound factors but also on adequate postoperative care, immobilization, and optimization of the wound environment [32,33].

The low complication profile in this study further supports the safety of skin graft reconstruction in appropriately selected patients. Postoperative infection, hypertrophic scar formation, and wound dehiscence were infrequent, and no major complications requiring surgical intervention, intensive management, or hospital readmission were observed in any patients. Patients who developed hypertrophic scars were managed with scar massage, topical betamethasone application, and silicone therapy. Betamethasone cream was massaged onto the scar and subsequently covered with silicone sheets for approximately 20 h daily for at least 6 months. These findings are clinically relevant because they suggest that skin graft reconstruction can achieve favorable early outcomes, even in a population with a substantial burden of diabetes and wound infection.

This study provides valuable institutional data from Oman and the wider Gulf region, where published data on complex wound reconstruction remain limited, despite the burden of trauma and metabolic comorbidities in this region. A major strength of this study was the inclusion of all eligible cases performed since the establishment of the Plastic Surgery Department at SQUH, providing early real-world data on local reconstructive practice. We performed this research study in the newly established plastic surgery department of our institution, providing baseline data for future quality improvement and comparative studies.

The present research study setting was not controlled; rather, we reported the standard grafting procedures followed in our department. The present research study in a small country like Oman clarifies and details how institutions make decisions about graft selection across various clinical contexts. To the best of our knowledge, this is the first report of data from Oman and the surrounding region.

However, this study has several limitations. The retrospective, single-center design and small sample size limited the generalizability of the findings. The cohort was heterogeneous with respect to wound etiology, anatomical site, depth, chronicity, wound-bed condition, and preoperative preparation. Although wound etiologies were reported separately, the small subgroup sizes prevented meaningful etiology-specific comparisons from being made. Wound size and defect surface area were not consistently documented, preventing adjustment for baseline wound severity and limiting the interpretation of graft choice, healing time, graft take, MatriDerm^®^ use, and complication risk. With only five partial graft-take events, the study was underpowered for predictor modelling; therefore, the reported associations should be considered exploratory, and the findings should be interpreted as descriptive institutional outcomes. MatriDerm^®^ was used in only five selected patients, precluding conclusions regarding comparative effectiveness. STSG and FTSG were selected according to clinical indication rather than standardized or randomized criteria, so direct comparison between graft types was not appropriate. Other clinically relevant variables, including bacterial burden, wound vascularity, and the timing and extent of debridement, were also not systematically available. Finally, the available follow-up limited robust assessment of long-term scar quality, functional outcomes, and reconstructive durability.

Future studies should focus on multicenter cohorts with larger sample sizes and longer follow-up periods to better define the predictors of graft success and evaluate long-term outcomes, including scar quality and functional recovery. Standardized outcome measures and patient-reported assessments may further improve our understanding of the clinical impact of skin graft reconstruction on complex wounds in the future. We firmly believe that standardized techniques for evaluating wound areas should be included in future studies.

## 5. Conclusions

In this descriptive institutional cohort study, a combined protocol of wound-bed preparation, skin graft reconstruction, and postoperative NPWT-assisted stabilization was associated with favorable early graft take and few complications. The findings should be interpreted cautiously because of wound heterogeneity, unavailable wound size measurements, and limited statistical power. Larger prospective multicenter studies incorporating standardized wound measurements are required to evaluate independent predictors of graft success.

## Figures and Tables

**Table 1:** Demographic data and comorbidities in the study population.

Variable	Category	n	%
Gender	Males	36	72
	Females	14	28
Age group	Pediatrics	5	10
	Adults	45	90
Diabetes	Yes	17	34
	No	33	66
Hypertension	Yes	13	26
	No	37	74
Dyslipidemia	Yes	7	14
	No	43	86
Hematological malignancy	Yes	5	10
	No	45	90
Hepatitis B	Yes	1	2
	No	49	98
Asthma	Yes	2	4
	No	48	96
Anemia	Yes	2	4
	No	48	96
Sickle cell disease	Yes	2	4
	No	48	96
Coronary artery disease	Yes	2	4
	No	48	96
Chronic kidney disease/End-stage renal disease	Yes	3	6
	No	47	94

**Table 2:** Distribution of wound etiologies among the study population (n = 50).

Category	Frequency	%
Trauma	14	28
Surgical/procedure-related	8	16
Burn	5	10
Infection/Necrotizing	11	22
Diabetic/metabolic ulcers	7	14
Oncological lesion-related	5	10

**Table 3:** Association between graft uptake on day 5–7 postoperatively and different factors.

		Complete Graft Take	Partial Graft Take	*p* Value
Age groups	Pediatrics	3	2	0.116
	Adults	42	3	
Diabetes	Yes	15	2	1.000
	No	30	3	
Infected Wound	Yes	25	3	1.000
	No	20	2	
Granulating Wound	Yes	11	1	1.000
	No	34	4	
Necrotized Wound	Yes	19	4	0.167
	No	26	1	

## Data Availability

The data supporting the findings of this study are available from the corresponding author upon reasonable request, subject to institutional and ethical restrictions.

## Abbreviations

The following abbreviations were used in this manuscript:

STSG	Split-thickness skin graft
FTSG	Full-thickness skin graft
NPWT	Negative-pressure wound therapy
VAC	Vacuum-assisted closure
BMI	Body mass index
IQR	Interquartile range
SQUH	Sultan Qaboos University Hospital

## References

[1] Frykberg R.G., Banks J. (2015). Challenges in the Treatment of Chronic Wounds. Adv. Wound Care.

[2] Labib A., Winters R. (2026). Complex Wound Management. StatPearls.

[3] Rasool A., Bashir S.A., Ahmad P.A., Bijli A.H., Baba U.F., Yasir M., Wani A.H. (2020). Management of Wounds with Exposed Bones or Tendons in Children by Vacuum-Assisted Closure Therapy: A Prospective Study. Indian J. Plast. Surg..

[4] Sen C.K. (2025). Human Wound and Its Burden: Updated 2025 Compendium of Estimates. Adv. Wound Care.

[5] Cai L., Hong Z., Zhang Y., Xiang G., Luo P., Gao W., Li Z., Zhou F. (2023). Management of Wounds with Exposed Bone Structures Using an Induced-membrane Followed by Polymethyl Methacrylate and Second-stage Skin Grafting in the Elderly with a 3-year Follow-up. Int. Wound J..

[6] Herskovitz I., Hughes O.B., Macquhae F., Rakosi A., Kirsner R. (2016). Epidermal Skin Grafting. Int. Wound J..

[7] Rijpma D., Claes K., Pijpe A., Hoeksema H., De Decker I., Verbelen J., Stoop M., De Mey K., Hoste F., Van Zuijlen P. (2025). Wound and Short-Term Scar Outcomes of Meek Micrografting Versus Mesh Grafting: An Intra-Patient Randomized Controlled Trial. Eur. Burn J..

[8] Samuel J., Gharde P., Surya D., Durge S., Gopalan V. (2024). A Comparative Review of Meshed Versus Unmeshed Grafts in Split-Thickness Skin Grafting: Clinical Implications and Outcomes. Cureus.

[9] Hilton C.M.H., Hölmich L.R. (2019). Full- or Split-Thickness Skin Grafting in Scalp Surgery? Retrospective Case Series. World J. Plast. Surg..

[10] Ramsey M.L., Walker B., Marietta M., Patel B.C. (2026). Full-Thickness Skin Grafts. StatPearls.

[11] Pérez-Guisado J., Fidalgo-Rodríguez F.T., Gaston K.L., Rioja L.F., Thomas S.J. (2012). [Skin graft, smoking and diabetes mellitus type 2]. Medicina.

[12] Abdulmughni L.A., Al-Sanabani J.A., Gilan W.M., Issa M.A., Jowah H.M. (2025). Split-Thickness Skin Graft Outcomes and Associated Risk Factors in Patients with Skin Defects at Al-Gumhouri Hospital, Sana’a, Yemen: A Prospective Observational Study. BMC Surg..

[13] Radzikowska-Büchner E., Łopuszyńska I., Flieger W., Tobiasz M., Maciejewski R., Flieger J. (2023). An Overview of Recent Developments in the Management of Burn Injuries. Int. J. Mol. Sci..

[14] Gamel M., Gael M., Bursztejn A.C. (2026). Skin Graft Bolstered by Negative Pressure Therapy in Chronic Wounds: A Systematic Review. J. Eur. Acad. Dermatol. Venereol..

[15] Jiang Z.-Y., Yu X.-T., Liao X.-C., Liu M.-Z., Fu Z.-H., Min D.-H., Guo G.-H. (2021). Negative-Pressure Wound Therapy in Skin Grafts: A Systematic Review and Meta-Analysis of Randomized Controlled Trials. Burns.

[16] Meo S.A., Sheikh S.A., Sattar K., Akram A., Hassan A., Meo A.S., Usmani A.M., Qalbani E., Ullah A. (2019). Prevalence of Type 2 Diabetes Mellitus Among Men in the Middle East: A Retrospective Study. Am. J. Men’s Health.

[17] Al-Maniri A.A.N., Al-Reesi H., Al-Zakwani I., Nasrullah M. (2013). Road Traffic Fatalities in Oman from 1995 to 2009: Evidence from Police Reports. Int. J. Prev. Med..

[18] Thourani V.H., Ingram W.L., Feliciano D.V. (2003). Factors Affecting Success of Split-Thickness Skin Grafts in the Modern Burn Unit. J. Trauma Inj. Infect. Crit. Care.

[19] Yammine K., Assi C. (2019). A Meta-Analysis of the Outcomes of Split-Thickness Skin Graft on Diabetic Leg and Foot Ulcers. Int. J. Low. Extrem. Wounds.

[20] Sun L., Patel A.J. (2021). Outcomes of Split vs Full-Thickness Skin Grafts in Scalp Reconstruction in Outpatient Local Anaesthetic Theatre. Scars Burns Heal..

[21] Braza M.E., Marietta M., Fahrenkopf M.P. (2026). Split-Thickness Skin Grafts. StatPearls.

[22] Kemp Bohan P.M., Cooper L.E., Fletcher J.L., Corkins C.J., Natesan S., Aden J.K., Carlsson A., Chan R.K. (2022). Impact of Dermal Matrix Thickness on Split-thickness Skin Graft Survival and Wound Contraction in a Single-stage Procedure. Int. Wound J..

[23] Mohamed M.M., Mohammed S.N., Abd El Salam A.M., Amin Abdel Gawad E.M. (2024). Study of the Influencing Factors and Their Predictive Value of Skin Graft Take in Early Excision and Grafting in Severe Burns: A Prospective Study. QJM Int. J. Med..

[24] Isitt C.E., McCloskey K.A., Caballo A., Sharma P., Williams A., Leon-Villapalos J., Vizcaychipi M.P. (2016). An Analysis of Surgical and Anaesthetic Factors Affecting Skin Graft Viability in Patients Admitted to a Burns Intensive Care Unit. Scars Burns Heal..

[25] Guogienė I., Kievišas M., Grigaitė A., Braziulis K., Rimdeika R. (2018). Split-Thickness Skin Grafting: Early Outcomes of a Clinical Trial Using Different Graft Thickness. J. Wound Care.

[26] Hahn H.M., Jeong Y.S., Lee I.J., Kim M.J., Lim H. (2022). Efficacy of Split-Thickness Skin Graft Combined with Novel Sheet-Type Reprocessed Micronized Acellular Dermal Matrix. BMC Surg..

[27] Laxmikantha C., Channasandra Shekar S., Srikantegowda M.H. (2025). Effectiveness of Split-Thickness Skin Grafting in Nonhealing Ulcers: Impact of Patient Demographics, Comorbidities, and Ulcer Etiology. Cureus.

[28] Ramanujam C.L., Han D., Fowler S., Kilpadi K., Zgonis T. (2013). Impact of Diabetes and Comorbidities on Split-Thickness Skin Grafts for Foot Wounds. J. Am. Podiatr. Med. Assoc..

[29] Haris S., Nema P.K., Charokar K., Gupta N. (2025). A Clinical Study of the Role of Split-Thickness Skin Autograft in Management of Wounds and Identification of Factors Influencing the Graft Uptake. Int. Surg. J..

[30] Holm S., Smith M.T.D., Huss F., Allorto N. (2024). Is the Preoperative Wound Culture Necessary Before Skin Grafting Minor Burns? A Pilot Study in a Low Resource Setting Burn Service. J. Burn Care Res..

[31] Bogari M., Budi A.S., Saputro I.D. (2023). Relationship between Split-Thickness Skin Graft and Hemoglobin Level in Burn Patients. Plast. Reconstr. Surg.-Glob. Open.

[32] Bovenberg M.S., Williams P.E., Goldberg L.H. (2024). Assessment of Pain, Healing Time, and Postoperative Complications in the Healing of Auricular Defects After Secondary Intent Healing Versus Split Thickness Skin Graft Placement. Dermatol. Surg..

[33] Markel J.E., Franke J.D., Woodberry K.M., Fahrenkopf M.P. (2024). Recent Updates on the Management of Split-Thickness Skin Graft Donor Sites. Plast. Reconstr. Surg.-Glob. Open.

